# Persistent Symptoms of Ganglion Cysts in the Dorsal Foot

**DOI:** 10.2174/1874325001711011308

**Published:** 2017-11-16

**Authors:** Akio Sakamoto, Takeshi Okamoto, Shuichi Matsuda

**Affiliations:** Department of Orthopaedic Surgery, Graduate School of Medicine, Kyoto University, Shogoin, Kawahara-cho 54, Sakyo-ku, Kyoto 606-8507, Japan

**Keywords:** Ganglion, Cyst, Pain, Artery, Nerve, Foot

## Abstract

**Background:**

A ganglion is a common benign cystic lesion, containing gelatinous material. Ganglia are most commonly asymptomatic, except for a lump, but symptoms depend on the location. A dorsal foot ganglion is typically painful. On the dorsal foot, the dorsalis pedis artery and the medial branch of the deep peroneal nerve are located under the fascia.

**Objective:**

Five female patients of average age 45.8 ± 20 years (range, 12 to 60 years) with a painful ganglion in the dorsal foot were analyzed.

**Results:**

Average lesion size was 2.94 ± 1.1 cm (range, 1.5 to 4.0 cm) and patients had experienced pain for a median of 2-3 years (range, 6 months to 3 years). Four patients had a single cystic lesion and 1 patient had developed multiple cystic lesions over the time that were associated with hypoesthesia. In 3 cases, symptomatic lesions were located deep beneath the fascia and were resected. In 2 cases, the depth of the non-resected lesions was shallow.

**Conclusion:**

The cause of a painful dorsal foot ganglion can be attributed to its location in the thin subcutaneous tissue over the foot bone, in addition to its proximity to a nearby artery and nerve. Mild symptoms caused by a dorsal foot ganglion seem to be persistent, and the deeper the location, the more likely is the need for resection. To avoid nerve injury, anatomical knowledge is prerequisite to any puncturing procedure or operation performed.

## 1 INTRODUCTION

A ganglion is a cystic lesion, characterized by mucinous contents, possibly connected to a joint capsule or a tendon sheath [1]. The wrist and the hand are sites particularly prone to ganglia, whereas the ankle and foot account for 11% of all cases [2]. One symptom of a ganglion is a lump which can vary in size and location [1]. In a previous study of 53 surgically resected ganglia of the foot and ankle, the mean width of the lesions was 2.7 cm, and 67.9% of patients reported related pain [2]. The reason ganglia tend to be symptomatic in the foot likely relates to their larger average size relative to wrist ganglia [3]. Reports of anatomically specific symptoms attributed to ganglia of the feet may be a consequence of the pain experienced when wearing shoes because of the added pressure and irritation created by the mass of the cyst [2].

The medial branch of the deep peroneal nerve runs distally on the dorsum of the foot lateral to the dorsalis pedis artery under the fascia [4]. The nerve innervates the area of the first interdigital cleft [5]. Symptoms associated with peripheral nerve involvement as a result of a ganglion in the dorsal foot have been reported [1, 6]. The current report describes 5 cases of ganglia in the dorsal foot; in 3 cases the ganglia were resected. All patients were symptomatic with regard to pain and one patient had neurological symptoms. Anatomical associations with the dorsalis pedis artery and the medial branch of the deep peroneal nerve were assessed as a possible cause of the pain for the purpose of providing appropriate treatment.

## 2 CASES

The clinical data are summarized in Table (**1**). From the consultation file, a total of 5 cases with a ganglion in the dorsal foot were found. The 5 cases were in 5 female patients with an average age of 45.8 ± 20 years (range, 12 to 60 years). Clinical signs and symptoms included a mass (lump) and pain, respectively, in all cases. The duration of the signs and symptoms ranged from 6 months to 3 years (median duration 2 and 3 years, respectively). There was no history of trauma or injury. One case reported numbness in the dorsal foot. On physical examination, hypoesthesia was confirmed in the area of the first interdigital cleft, where the medial branch of the deep peroneal nerve was associated.

For all cases, plain radiographs revealed no abnormalities of the foot. The diagnosis of a ganglion was made based upon a physical examination and magnetic resonance imaging (MRI) findings. MRI findings revealed a fibrous cystic lesion containing fluid material in all cases. The cystic lesion was located over the talus to the navicular (1 case), the cuneiform to the metatarsus (1 case), solely the cuneiform (2 cases), and the talus and the cuneiform-metatarsus for the case with multiple cystic lesions Fig. (**1**). The signal intensity on the MRI for the interior of the lesions was consistent in all cases with homogenous low signal intensity on T1-weighted images and a homogenous high signal intensity on T2-weighted images. The cystic wall was characterized by low signal intensity on T1- and T2-weighted images. The sizes of the ganglia measured using the MRI ranged from 1.5 to 4.0 cm, with an average diameter of 2.94 ± 1.1 cm. The lesions were on the right side in 4 cases, and on the left side in one case. In 3 cases, the lesions were located under the fascia Fig. (**2**), and in 2 cases the lesions were above the fascia Fig. (**3**). A singular nodule was present in 4 cases, whereas multiple cystic lesions were observed in 1 case.

Conservative treatment was recommended initially, except for the case with neurological symptoms. When requested by patients, a resection was performed on lesions with continuous symptoms lasting longer than 6 months. Eventually, 3 lesions were surgically resected, all of which were located deep under the fascia. During surgery, these cysts were carefully dissected from the artery and the nerve, using loupes. Histologically, the cystic wall was fibrocollagenous tissue with focal areas of myxoid change, characteristic of ganglion in each case. Adverse side effects of a neurological nature were not observed after the procedure for the 3 cases that underwent resection. Symptoms of pain disappeared after the resection. The 2 patients with lesions located over the fascia did not undergo surgical resection and reported persistent pain.

## 3 DISCUSSION

A ganglion is a cystic lesion containing mucinous materials. The diagnoses of a ganglion or ganglia in all the current cases were made following a physical examination and based on MRI findings that were consistent with the characteristics of a cystic lesion; that is, low-signal intensity on T1-weighted images and high signal intensity on T2-weighted images. MRIs also revealed the anatomical association of each ganglion with surrounding structures such as the vessels and the nerves.

In our series, all 5 patients reported pain in the dorsal foot and the size of the lesions ranged from 1.5 to 4.0 cm, with an average width of 2.9 cm. The lesions were generally flat-shaped with a thickness of less than 1 cm. The intensity of symptoms seemed to be related not only to the size of the ganglia [3], but also their specific location in the dorsal foot. The symptoms seemed to be related to the thin subcutaneous tissue over the foot bone, and all lesions were close to the dorsalis pedis artery and the medial branch of the deep peroneal nerve.

One among 5 of our cases had neurological symptoms which were associated with the medial branch of the deep peroneal nerve. Peripheral nerve symptoms due to a ganglion are rare [7-9]. The mechanisms causing the peripheral nerve symptoms related to ganglia have been reported to be compression, stretching, and friction [10]. A major cause of foot ganglion symptoms is associated with the wearing of shoes [2, 3]. Among 53 foot ganglion cases, 27 cases were located in the dorsal foot [2]. Two cases of dorsal foot ganglia causing neurological symptoms have been previously reported, in which the symptoms of pain were attributed to deep peroneal nerve involvement, similar to what occurred in the current case [1, 6]. The neurological symptoms in our current case had a working diagnosis of an intraneural ganglion, although the association with the medial branch of the deep peroneal nerve seemed obscure based on the MRI. Intraneural ganglia are unusual benign mucoid-containing cysts within the epineurium of the peripheral nerves [11]. The majority of these types of cases involve a lower extremity and affect the common peroneal nerve and its branches [12, 13]. Intraneural ganglia arise from the joint and spread to the nerve *via* articular branching, similar to ganglia [14, 15].

The treatment strategy for ganglia is conservative and includes aspiration [2]. When the lesion is recurrent or painful, surgical excision is recommended [2, 16]. In our series, 3 out of the 5 patients underwent resection. The lesions were deeply located in cases that opted for resection. The deep location might contribute to the rather intense symptoms experienced, thus making it necessary to resect the dorsal foot ganglia. Nerve injury during ganglion resection procedures in the ankle and foot represents an adverse side effect, and cutaneous nerve injury occurs in 10% of resections [17]. During dorsal foot ganglion resection procedures, paying attention to the medial dorsal cutaneous nerve is extremely important. The nerve is located over the fascia, and governs sensations in most of the dorsum of the foot [5].

## CONCLUSION

If ganglia develop in the foot, they are often symptomatic. We assume that the location of ganglia in the dorsal foot is a typical cause of persistent pain. In the current report, a small number of cases (5 in total) of ganglia in the dorsal foot were described retrospectively. Symptoms are generally mild, but in cases with a deeply located ganglion that presents with more intense pain and other neurological symptoms, resection may be necessary. In such cases, the availability of detailed anatomical data regarding the location and association of ganglia with other structures is recommended before any puncture or resection procedures to avoid potential nerve injury.

## ABBREVIATION

MRI: Magnetic resonance imaging

## Figures and Tables

**Fig. (1):**
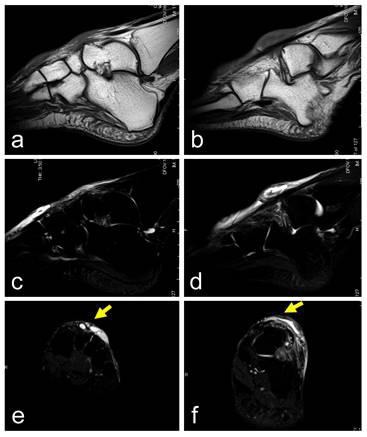
A 60-year-old female with a ganglion in the dorsal foot. The cystic lesions, with low signal intensity on a T1-weighted image (**a–b**) and high signal intensity on T2-weighted images (**c–f**), are located over the cuneiform to metatarsal bones (**a, c, e**) and the talus (**b, d, f**). The dorsalis pedis artery is located under the lesion (yellow arrows; **e, f**). T2-weighted images with fat-suppression (**c-f**). Sagittal views (**a-d**) and axial views (**e–f**).

**Fig. (2):**
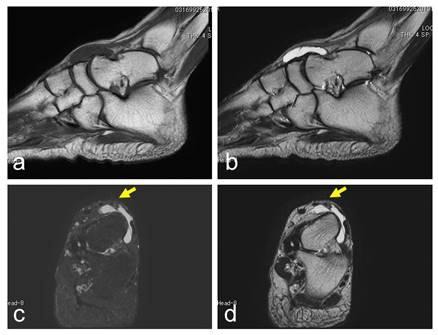
A 55-year-old female with a ganglion in the dorsal foot. A cystic lesion, with low signal intensity on a T1-weighted image (**a**) and high signal intensity on a T2-weighted image (**b–d**) located over the talus to the navicular (**a–b**). The dorsalis pedis artery is located over the lesion (yellow arrows; **c–d**). A T2-weighted image with fat-suppression (**c**). Sagittal views (**a–b**) and axial views (**c–d**).

**Fig. (3):**
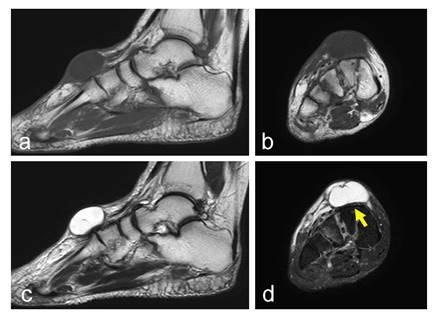
A 66-year-old female with a ganglion in the dorsal foot. The lesion, with low signal intensity on a T1-weighted image (**a–b**) and high signal intensity on a T2-weighted image (**c–d**), is shallowly located over the cuneiform. The dorsalis pedis artery is located below the lesion (yellow arrows; **d**) A T2-weighted image with fat-suppression (**d**). Sagittal views (**a, c**) and axial views (**b, d**).

**Table 1:** Clinical summary of dorsal foot ganglia.

Case	Age, Gender	Side	Size (width)	Symptom	Term	Bone(s)	Lesion depth	Artery in Relation to Fascia	Peroneal Nerve in Relation to Fascia	Resection
1	55, F	L	4 cm	pain	3 y	*talus*-*navicular*	deep	above	above	+
2	12, F	L	1.5 cm	pain	2–3 y	*cuneiform-metatarsus*	deep	above	above	+
3	60, F	L	3 cm (multiple)	Pain and numbness	6 mo	*talus,* *cuneiform-metatarsus*	shallow	under	under	–
4	42, F	L	2.2 cm	pain	2–3 y	*cuneiform*	deep	side	side	+
5	66, F	R	4 cm	pain	1 y	*cuneiform*	shallow	under	under	–

Artery: dorsalis pedis artery, F: female, Peroneal N: medial terminal branch of deep peroneal nerve, L: left, M: male, mo: month, R: right, y: year.
